# Synthesis of Rare-Earth Nanomaterials Ag-Doped NaYF_4_:Yb^3+^/Er^3+^@NaYF_4_:Nd^3+^@NaGdF_4_ for In Vivo Imaging

**DOI:** 10.3390/nano12050728

**Published:** 2022-02-22

**Authors:** Wei Zhang, Yang Zang, Yanli Lu, Jinghui Han, Qingyun Xiong, Jinping Xiong

**Affiliations:** 1 Beijing Key Laboratory of Electrochemical Process and Technology of Materials, Beijing University of Chemical Technology, Beijing 100029, China; 2019200387@mail.buct.edu.cn (W.Z.); 2019310030@mail.buct.edu.cn (Y.Z.); 2019310036@mail.buct.edu.cn (Y.L.); 2020200494@mail.buct.edu.cn (Q.X.); 2 State Key Laboratory of Organic-Inorganic Composites, Beijing University of Chemical Technology, Beijing 100029, China; 2021400008@buct.edu.cn; 3 College of Ecology and Resources Engineering, Wuyi University, Wuyishan 354300, China

**Keywords:** upconversion, near-infrared, intensity, in vivo imaging

## Abstract

In this study. a novel near-infrared fluorescent-driven contrast agent (Ag-doped NaYF_4_:Yb^3+^/Er^3+^@NaYF_4_:Nd^3+^@NaGdF_4_) was synthesized using a coprecipitation-hydrothermal-solvothermal-solvothermal (CHSS) method. The results shows that hexagonal NaYF_4_:Yb^3+^/Er^3+^ with a diameter of 300 nm was successfully synthesized by the CHSS method. The new contrast agent was characterized using scanning electron microscopy, fluorescence spectrometry, transmission electron microscopy, energy-dispersive spectrometry and ultraviolet-visible light diffuse reflectance absorption spectroscopy. Even at low concentrations (0.2 M), this proposed contrast agent can be excited by near-infrared light with a wavelength of 980 nm and emits a dazzling green light with a wavelength of 540 nm, and the comparison of the luminescence intensity proves that doping with silver increases the luminescence intensity of the upconverted nanomaterial by nearly 13 times based on the calculated quantum yield. TEM images show the successful preparation of silver nanoparticles with a diameter of 30 nm, and the energy spectrum shows the successful doping of silver nanoparticles and the successful preparation of the core-shell structure of NaYF_4_:Yb^3+^/Er^3+^@NaYF_4_:Nd^3+^@NaGdF_4_. Furthermore, the mechanism of the increased luminous intensity has been studied using simulation calculations. Finally, cytotoxicity tests were used to test material which was modified by 1,2-distearoyl-sn-glycero-3-phosphoethanolamine-N-[amino(polyethylene glycol)-2000] (DSPE-PEG_2K_), and the biocompatibility was significantly improved, meeting the standard for biological applications.

## 1. Introduction

Upconverted rare-earth nanomaterials are commonly used in various fields because of their high fluorescence intensity. Recently, their application in biomedicine has been widely studied. Many scholars have proposed that they can be used in biological imaging because they offer significant advantages in the fight against major diseases, such as cancer [1,2,3]. However, a common difficulty is that they have insufficient luminous intensity and are toxic to biological cells, so it is necessary to modify their structure and modify their surface [4]. To mitigate this problem, several imaging probes were combined into a single multi-modality imaging system, even with some considerable restrictions such as the need to use a sophisticated synthetic processes and the heterogeneity of the resulting nanostructures. As a result of their enhanced optical and magnetic properties, and also improved X-ray attenuation, lanthanide-doped upconverted nanoparticles (UCNPs) might be perfect for building multifunctional bioprobes by doping with various rare earth ions without modifying other functions. Many scholars have proposed doping Mo^3+^, Cu^2+^, and other metal ions in the NaYF_4_:Yb^3+^/Er^3+^ unit cell to increase the luminous intensity, but the effect is not significant [5,6]. Others have proposed doping with silver, which also has a significant effect, but silver has a high light-to-heat conversion efficiency, can cause cell apoptosis without targeting, and cannot be used in biology [7,8]. Other scholars have proposed constructing core-shell structures such as NaYF_4_:Yb^3+^/Er^3+^@NaGdF_4_:Yb^3+^, NaYF_4_:Yb^3+^/Er^3+^@NaNdF_4_:Yb^3+^/Tm^3+^@NaGdF_4_:Yb^3+^ [9,10,11]. Alternatively, the reverse microemulsion method can be used to construct a layer of silica or porous silica, such as NaYF_4_:Yb^3+^/Er^3+^@SiO_2_, NaYF_4_:Yb^3+^/Er^3+^@NaGdF_4_:Yb^3+^@m-SiO_2_ [12,13,14]. Although these operations can reduce the biological toxicity and meet the basic requirements for use in biological cells or animals, there is no doubt that these changes will reduce the luminous intensity of the upconverted luminescent materials [15,16,17]. If such a material is used as a contrast agent for in vivo imaging, the image will be unclear, the tumor cannot be observed, and further diagnosis and treatment will be difficult [18,19,20]. Unfortunately, when these materials meet the biological requirements, their luminous intensity will inevitably be reduced, so that imaging cannot be performed to obtain a clear image [21,22]. Considering the high desire to develop UCNP nanomaterials with highly effective imaging capability as well as high biocompatibility to prevent apoptosis or biological organ failure, UCNPs doped with sliver nanoparticles are an ideal candidate because they are easy to fabricate, have enhanced luminescence, and their surface is easily modified [23,24]. More notably, following illumination, the UCNPs are harmless to normal tissues but cytotoxic to malignant ones [25]. To the best of our knowledge, however, there appears to be no mention in the literature of methods to create theranostic nanoplatforms integrating multi-modality bioimaging with light triggered chemotherapy.

**Scheme 1:**
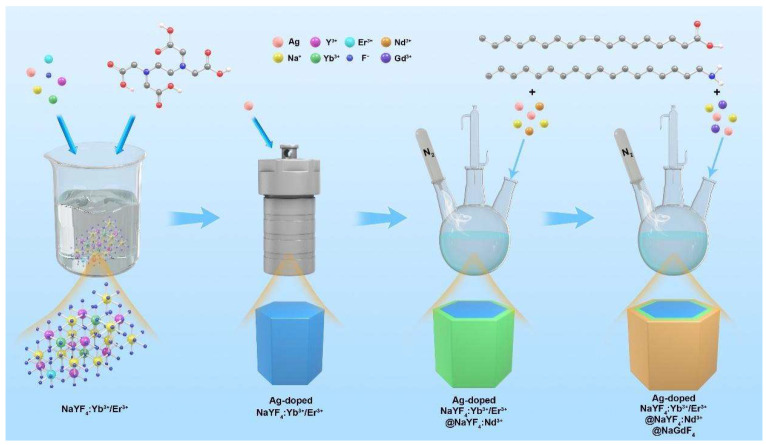
Flow chart of the CHSS method synthesis of Ag-doped NaYF_4_:Yb^3+^/Er^3+^@NaYF_4_:Nd^3+^@NaGdF_4_.

Therefore, this paper proposes a new upconverted nanomaterial structure that not only has extremely low cytotoxicity and good luminescence intensity, but also good targeting that can accurately label tumor cells and can be used for in vivo imaging, and obtain clear tumor images instead of requiring expensive CT, MRI, and other medical equipment. This study proposes the use of a coprecipitation-hydrothermal-solvothermal-solvothermal (CHSS) method to synthesize the core-shell structure of Ag- NaYF_4_:Yb^3+^/Er^3+^@NaYF_4_:Nd^3+^@NaGdF_4_. Theis shell structure is different from structures presented by other scholars mentioned above, and it will not sacrifice the luminous intensity to improve its biocompatibility. Scheme 1 shows the entire CHSS method process.

## 2. Experimental

### 2.1. Materials

Y_2_O_3_ (99.99%), Nd_2_O_3_ (99.99%), Gd_2_O_3_ (99.99%), Yb_2_O_3_ (99.99%), Er_2_O_3_ (99.99%), nitric acid (68%), sodium fluoride (99.99%), citric acid (99.99%), chloroform (99.5%), cyclohexane (99.5%), oleic acid (≥95%), octadecylamine (≥95%), silver nitrate (99.99%), ethylene diamine tetraacetic acid (EDTA, ≥99%), sodium hydroxide (≥98%), 1,2-distearoyl-sn-glycero-3-phosphoethanolamine-N-[amino(polyethylene glycol)-2000] (DSPE-PEG_2K_) and poly(vinylpolypyrrolidone) (PVP, average molecular weight of 1,000,000–1,500,000) were purchased from Aladdin (Shanghai, China). Cell counting kit 8 (CCK-8), phosphate-buffered saline (PBS, pH = 6.8), trypsin-EDTA (80%), and fetal calf serum (10%) were purchased from BOVOGEN (Shanghai, China). All the chemicals were used as received without further purification.

### 2.2. Synthesis of Sliver Nanoparticles

Sixty mL of 0.05 mol/L of citric acid solution was added dropwise to 3 mL of 0.02 mol/L of AgNO_3_ to obtain a mixture. After 5 min of continuous stirring, the solution was transferred to a 100 mL reactor and placed in an oven for the reaction to complete at 120 °C for 6 h. The reaction mixture was then cooled to room temperature, washed, and centrifuged to obtain solid Ag nanoparticles, added to 10 mL of deionized water and PVP, and then placed in a test tube to prepare the sol for use.

### 2.3. Synthesis of Ag-NaYF_4_:Yb^3+^/Er^3+^

RE_2_O_3_ (RE = Y, Yb, Er) was heated in excess nitric acid to achieve complete dissolution and then transferred to a vacuum system for evaporation to obtain a solid RE(NO_3_)_3_, which was then dissolved in deionized water and recrystallized twice. A certain amount of solid RE(NO_3_)_3_ was dissolved in deionized water, and EDTA (molar ratio of EDTA: RE(NO_3_)_3_ = 1:1) was added and stirred at 600 rpm for 1 h, the mixture was then weighed and mixed in sodium fluoride in deionized water by ultrasound, and then the solution was added and stirred at 600 rpm for 1 h. Finally, the pH value was adjusted to 5.5 with NaOH and add 10 mL of silver dispersion, and then place it in a hydrothermal kettle to react at 190 °C for 24 h. The reaction products were cooled, centrifuged, and washed twice with ethanol/deionized water (1:1 *v*/*v*), and dried in vacuum at 80 °C for 3 h. The resultant powder is dispersed in cyclohexane for later use.

### 2.4. Synthesis of Ag- NaYF_4_:Yb^3+^/Er^3+^@ NaYF_4_:Nd^3+^

First, 0.5 mmol Ln (NO)_3_ (Ln = Y, Nd) was dissolved in 1 mL of water, 2 mmol sodium fluoride was dissolved in 4 mL water, and 1.2 g sodium hydroxide was weighed into a 50 mL single-mouth flask, and 4 mL water was added to dissolve it completely. Nine mL of anhydrous ethanol and 20 mL oleic acid were added, stirring for approximately 10 min, then Ln (NO)_3_ aqueous solution and sodium fluoride aqueous solution were added one by one, stirred for approximately 1 h, and the same amount of ethanol was added to precipitate the product, which was centrifuged for 10 min at 10000 rpm, then washed once with ethanol and cyclohexane. The final product was dispersed in 2 mL of cyclohexane.

Octadecene (10 mL) and oleic acid (6 mL) were added to a 100 mL three-mouth flask, then 2 mL of NaREF_4_ (RE = Y, Yb, Er) dispersion in cyclohexane was added, stirred for 30 min, then 10 mL octadecene, 6 mL oleic acid, and 2 mL of cyclohexane dispersion containing 0.5 mmol NaLnF_4_ (Ln = Y, Nd) nanoparticle precursor was added, under the protection of nitrogen, stirred at 70 °C for 30 min to remove cyclohexane in the system, while heating at 10 °C/min to 280 °C during the heating process. A condenser was added when the temperature reached 200 °C, and the reaction mixture was cooled to room temperature after 1 h. Centrifugation was performed at 10,000 rpm for 5 min to collect the precipitate, which was then washed with ethanol and finally dispersed in cyclohexane.

### 2.5. Synthesis of Ag- NaYF_4_:Yb^3+^/Er^3+^@ NaYF_4_:Nd^3+^@NaGdF_4_

First, 0.5 mmol Gd (NO)_3_ was dissolved in 1 mL of water, 2 mmol sodium fluoride was dissolved in 4 mL water, and 1.2 g sodium hydroxide was weighed into a 50 mL single mouth flask, and dissolved completely in 4 mL water. Nine mL of anhydrous ethanol and 20 mL oleic acid were added, stirred for approximately 10 min, then Ln (NO)_3_ aqueous solution and sodium fluoride aqueous solution were added one by one, stirred for approximately 1 h, then the same amount of ethanol was added to precipitate the product, which was centrifuged for 10 min at 10,000 rpm, then wash once with ethanol and cyclohexane. The final product was dispersed in 2 mL cyclohexane.

After adding 10 mL octadecene and 6 mL oleic acid to a 100 mL three-mouth flask, then 2 mL NaREF_4_ (RE = Y, Yb, Er) @NaLnF_4_ (Ln = Y, Nd) solution in cyclohexane was added, stirring for 30 min, then 10 mL octadecene, 6 mL oleic acid, and 2 mL of 0.5 mmol cyclohexane solution of NaGdF_4_ nanoparticle precursor was added, under the protection of nitrogen, stirred at 70 °C for 30 min to remove cyclohexane in the system, while heating at 10 °C/min to 280 °C, during the heating process. A condenser was added when the temperature reached 200 °C, and the reaction mixture was cooled to room temperature after 1 h. Centrifugation was performed at 10,000 rpm for 5 min to collect the precipitate, which was washed again with ethanol, and finally dispersed in cyclohexane.

### 2.6. DSPE-PEG_2K_ modified Ag- NaYF_4_:Yb^3+^/Er^3+^@ NaYF_4_:Nd^3+^@NaGdF_4_

First, 6 mL of NaYF_4_:Yb^3+^/Er^3+^@ NaYF_4_:Nd^3+^@NaGdF_4_ rare-earth nanocrystals (0.4 mmol) dispersed in chloroform were mixed with 20 mL of DSPE-PEG_2K_ (100 mg) chloroform solution in 5 mL open glass bottles. After heating at 75 °C for 5 min to remove the chloroform, 24-mL of water was added to complete the ultrasonic dispersion, stirring at 75 °C for 10 min, cooling to room temperature, centrifuging at 18,000 rpm for 8 min to collect the precipitate, and adding 1 mL of normal saline to disperse it. Large particles were removed by centrifugation at 5000 rpm for 5 min and then dried by a blast of nitrogen at 75 °C.

### 2.7. Characterization

Transmission electron microscopy (TEM) measurements were performed on a 2011 microscope (JEOL, Beijing, China) operating at 200 kV. All samples were first dispersed in ethanol and then collected using a Cu grid covered with a carbon film for measurement. To determine the elemental composition of the samples, energy-dispersive X-ray spectroscopy (EDS) of the samples was performed on a JEOL 2010 EDS instrument using high-resolution transmission electron microscopy (HRTEM) measurements. Inductively coupled plasma-atomic emission spectrometry (ICPAES) was performed using a 7300DV apparatus (Perkin Elmer, Shanghai, China). Scanning electron microscopy (SEM) images were obtained using an XL30 electron microscope (Philips, Eindhoven, Netherlands) operating at 20 kV. Before this characterization, a Au film was sprayed on the sample. The upconversion luminescence spectrum was obtained using a spectrum analyzer (ANDO AQ6317, Yokohama, Japan). The sample was placed in a 1.0-cm path length support, which was excited using a 980-nm CW semiconductor diode laser (P_max_ 800 mW, 1000 mA). The upconversion luminescence spectrum was obtained by the spectrophotometer using a multimode fiber having a core diameter of 0.6 mm. The distance between the top of the fiber and sample is ~2 mm.

### 2.8. First-Principles Calculations

First-principles calculations were performed using spin-polarized density functional theory (DFT) with generalized gradient approximation of Perdew-Burke-Ernzerhof implemented in the Vienna Ab-Initio Simulation Package. The valence electronic states were expanded using plane waves with the core-valence interaction represented by the projector augmented plane wave method and a cutoff of 500 eV. For the optimization of the equilibrium geometries of NaYF_4_ and RE-doped NaYF_4_ compounds, Brillouin zone integration was sampled with 3 × 3 × 5 k-grid mesh. The structures were completely relaxed until the maximum force on each atom becomes less than 0.02 eV/Å.

### 2.9. CCK-8 Assay for Cytotoxicity

The culture medium in the flask was sucked out, washed with PBS, and then 0.25% of trypsin was added to digest cells after culturing HeLa cells in the logarithmic growth phase. After the removal of trypsin, the DMEM medium containing 10% fetal bovine serum was added to blow the cells, which were then transferred to the sampling tank and blown well. Subsequently, 100 µL cells were injected into a 96-well plate (1 × 10^4^ cells/well) and incubated for 24 h in a constant temperature incubator at 37 °C (5% CO_2_). The cells were incubated for 6 h in an incubator at 37 °C with 5% CO_2_ in accordance with concentrations of 200, 300, 400, 500, and 600 µg/mL. The culture medium was blotted out, PBS was rinsed twice, the culture medium was replaced in the 96-well plates with 100 µL of fresh DMEM containing 10% fetal bovine serum, and then 10 µL of CCK-8 solution was added to each well. The absorbance of each well at 450 nm was measured using a microplate reader after 2 h of culturing in the incubator.

### 2.10. In Vivo Imaging

A mouse tumor model was constructed by subcutaneous injection of HeLa cells through the thigh. After 14 days of culture, we intratumorally injected Ag- NaYF_4_:Yb^3+^/Er^3+^@ NaYF_4_:Nd^3+^@NaGdF_4_-DSPE-PEG_2K_ into the tumor site and imaged them by a modified in vivo Maestro whole-body imaging system (Berthold Technologies NightOWLIILB983, Bad Wildbad, Germany). A strong in vivo signal began to arise from the location where Ag- NaYF_4_:Yb^3+^/Er^3+^@ NaYF_4_:Nd^3+^@NaGdF_4_-DSPE-PEG_2K_ was injected, but no autofluorescence was detected elsewhere.

## 3. Results and Discussion

**Figure 1:**
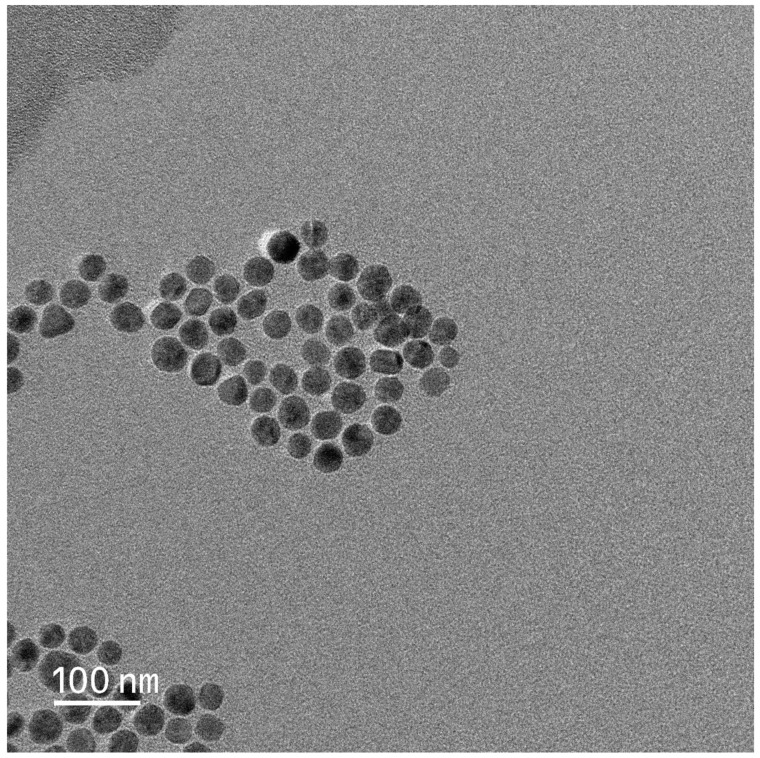
TEM image of Ag nanoparticles.

**Figure 2:**
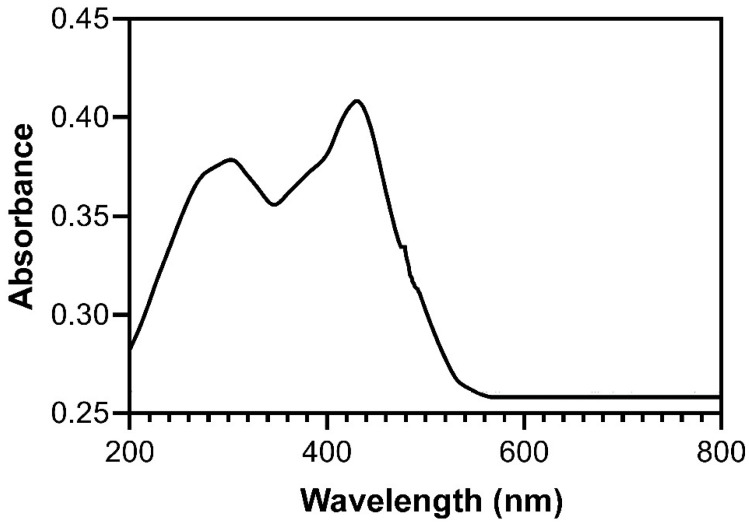
UV absorption spectrum of Ag nanoparticles.

The TEM images of Ag nanoparticles (Figure 1) prepared using the hydrothermal method show that they are spherical and have an average diameter of ~30 nm. The UV-visible absorption spectra of Ag nanoparticles (Figure 2) obtained using a UV absorption spectrophotometer shows that the absorption peak of Ag nanoparticles is located at 430 nm, which is consistent with the results reported in the literature.

**Figure 3:**
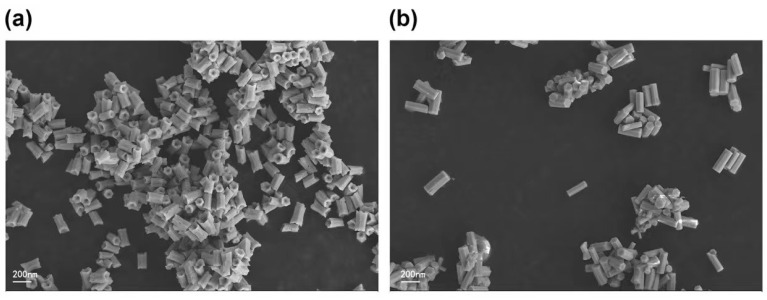
SEM image of UCNPs (**a**) and Ag-UCNPs (**b**).

**Figure 4:**
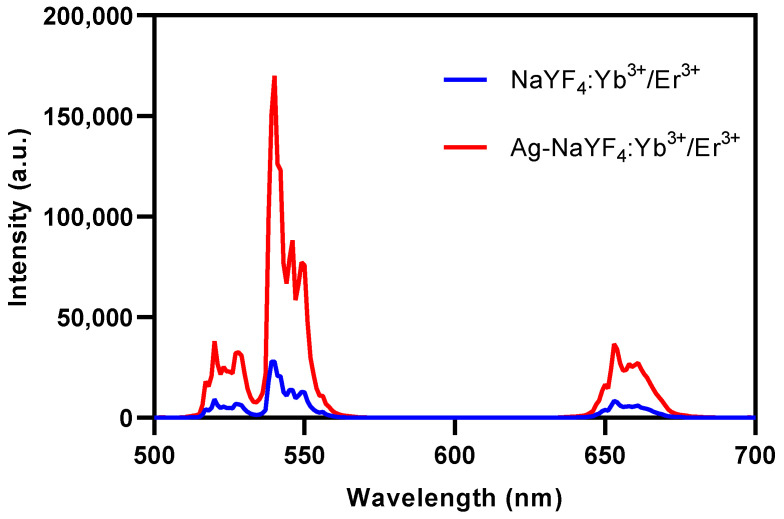
Luminescent intensity of NaYF_4_:Yb^3+^/Er^3+^ and Ag-NaYF_4_:Yb^3+^/Er^3+^.

**Figure 5:**
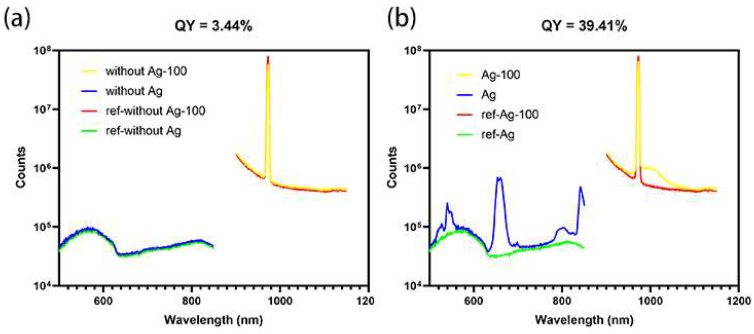
Upconversion room temperature quantum yield, (**a**): Quantum yield of NaYF_4_:Yb^3+^/Er^3+^ is 3.44%, (**b**): Quantum Yield of Ag-NaYF_4_:Yb^3+^/Er^3+^ is 39.41%.

SEM shows the normal hexagonal crystal of NaYF_4_:Yb^3+^/Er^3+^ (Figure 3a) has defects on both ends and sides, whereas the hexagonal crystal of Ag-NaYF_4_:Yb^3+^/Er^3+^ (Figure 3b) doped with silver is smooth. The reason for this change is that the doping of silver nanoparticles changes the structure of NaYF_4_:Yb^3+^/Er^3+^, some silver atoms are protonated to replace part of Er^3+^, and the remaining silver atoms are distributed inside the NaYF_4_:Yb^3+^/Er^3+^ unit cell. An interstitial solid solution is formed, causing the NaYF_4_:Yb^3+^/Er^3+^ inner space to be reduced and squeezed to the outside and contracted by surface force to fill the defects on the surface and both ends. This smooth crystal structure increases luminous intensity. This view was confirmed by comparing their luminous intensities. Note that NaYF_4_:Yb^3+^/Er^3+^ and Ag-NaYF_4_:Yb^3+^/Er^3+^ were prepared into 0.2 M solutions and their luminescence intensity (Figure 4) at 980 nm wavelength was tested, respectively. The results showed that the luminescence intensity increased by nearly 6.5 times after doping with sliver. The quantum yield before and after doping with silver nanoparticles was tested (Figure 5), and the results showed that the doping of silver nanoparticles improved the quantum yield nearly 13-fold. This phenomenon can be explained by first-principles calculations.

The effect mechanism of silver nanoparticle doping on the luminescence of NaYF_4_:Yb^3+^/Er^3+^ is as follows: In the luminescent system, the photoelectron transitions of ^1^I_6_ → ^3^H_6_, ^1^I_6_ → ^3^F_4_ and ^1^D_2_ → ^3^H_6_ generate luminescence at ~292 nm, ~345 nm and ~362 nm, respectively. UV emission; photoelectron transitions of ^1^D_2_ → ^3^F_4_ and ^1^G_4_ → ^3^H_6_ result in blue emission at ~450 nm and ~475 nm. In the non-silver-doped NaYF_4_:Yb^3+^/Er^3+^ light-emitting system, the Yb^3+^ ions first undergo ^2^F_5/2_ → ^2^F_7/2_ (Yb^3+^) photon transition under the excitation of 980 nm near-infrared laser, and then the photoelectrons reach the excited state of Er^3+^ energy level through 3–5 photon process. The re-transition of photogenerated electrons from the excited state to the ground state will generate up-conversion emission light in 5 bands, and the corresponding energy theory is as follows:

(1) Three-photon process

The electron of Er^3+^ accepts the energy of the first photoelectron pumping transition and then transitions (^3^H_6_ → ^3^H_5_), and rapidly relaxes to the ^3^F_4_ energy level (^3^H_5_ → ^3^F_4_) without radiation, and continues to accept the second photoelectron transition Energy, excited to ^3^F_2_ energy level, no radiation relaxation to ^3^H_4_ energy level (^3^F_4_ → ^3^H_4_), accept the third photoelectron transition energy, excited to ^1^G_4_ energy level, ^3^H_4_ → ^1^G_4_ (Er^3+^), fall back to ground state ^3^H_6_, emission 476 nm light.

(2) Four-photon process

The photoelectron at the ^1^G_4_ level accepts the 4th photoelectron pump transition energy, which is excited from the ^1^G_4_ level to the ^1^D_2_ level (^3^H_4_ → ^1^D_2_). If it falls back from ^1^D_2_ to the ^3^F_4_ energy level, it emits light at 450 nm; if it falls back from ^1^D_2_ to the ground state ^3^H_6_, it emits light at 345 nm, and the spectrum shows that the emission light at this wavelength is the strongest.

(3) Five-photon process

The photoelectron of the ^1^D_2_ energy level accepts the fifth photoelectron pumping transition energy and is excited from the ^1^D_2_ energy level to the ^3^P_2_ energy level (^1^D_2_ → ^3^P_2_), and then relaxes to the 1I6 energy level (^3^P_2_ → ^1^I_6_) without radiation, and then the excited state of Er^3+^ The electron transitions to the ground state (^1^I_6_ → ^3^H_6_) and emits light at 292 nm.

**Figure 6:**
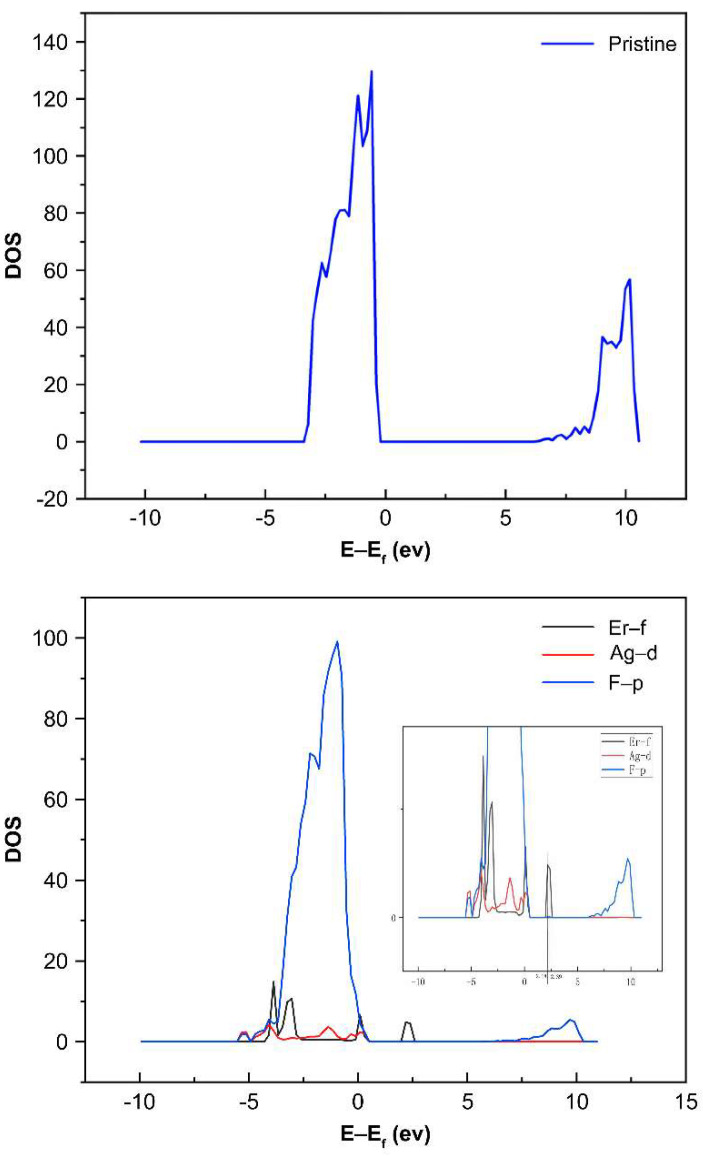
Computed DOS of the perfect NaYF_4_ and Ag-NaYF_4_ doping structures.

DFT calculations were performed to better understand the electronic properties and chemical origins of sliver nanoparticles enhancement. We chose Ag-NaYF_4_ to investigate the effect of sliver nanoparticles on the Er luminescence center of materials because of the difficulty of convergence in the multi-rare earth-doping systems. As shown in Figure 6, the calculated total density of states (DOS) shows a significant bandgap in pure NaYF_4_. The bandgap is 6.78 eV in size, which corresponds to the experimental values. The bandgap narrows and more hybridized electronic states occur near the Fermi level in Ag-NaYF_4_ when sliver nanoparticles are doped in Er-NaYF_4_, (Figure 6). The hybridized electronic states near the Fermi level are shown to be contributed by the 4f orbital of Er’s 4d orbital of Ag and the 2p orbital of F. The enhancement of the total DOS near the Fermi level in Ag-NaYF_4_ may make the system easier to absorb the photons and enhance luminescence.

**Figure 7:**
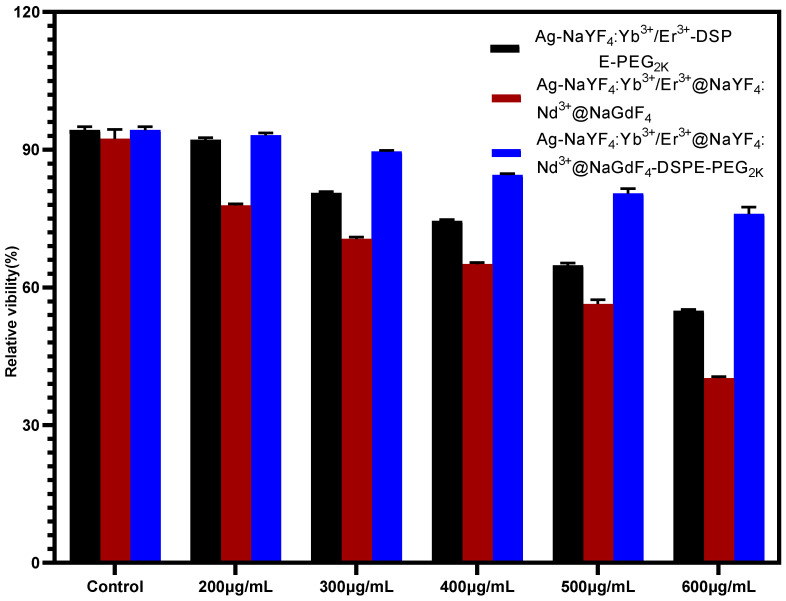
Cytotoxicity of different concentrations of Ag-NaYF_4_:Yb^3+^/Er^3+^@ NaYF_4_:Nd^3+^@NaGdF_4_ before and after modified by DSPE-PEG_2K_ and Ag-NaYF_4_:Yb^3+^/Er^3+^-DSPE-PEG_2K_.

**Figure 8:**
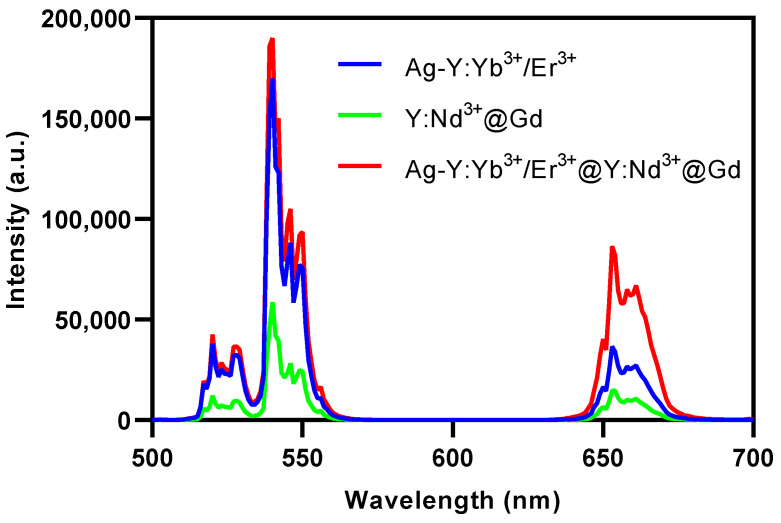
Luminescent intensity of Ag-NaYF_4_:Yb^3+^/Er^3+^, NaYF_4_:Nd^3+^@NaGdF_4_ and Ag-NaYF_4_:Yb^3+^/Er^3+^@ NaYF_4_:Nd^3+^@NaGdF_4_.

Although this luminescence intensity is sufficient for near-infrared imaging of organisms, it does not solve the problem of biotoxicity (Figure 7). We chose NaYF_4_:Nd^3+^@NaGdF_4_ to coat Ag-NaYF_4_:Yb^3+^/Er^3+^ because one can perform surface modifications better. Surprisingly, we found that the NaYF_4_:Nd^3+^@NaGdF_4_ structure also had a fluorescence effect and showed more excellent luminescence intensity after it was coated with Ag-NaYF_4_:Yb^3+^/Er^3+^ (Figure 8). This removes the constraint that the luminescent material covered by the shell will reduce the luminous intensity. Under normal conditions, the shell material tends to block the near-infrared penetration, which decreases the luminous intensity, and the increase in the luminous intensity observed here is because the shell material is excited to release photons to compensate for the reduced luminous intensity. The modified rare-earth nanomaterials were dispersed in normal saline to prepare different concentrations, after which HeLa cells were cultured for 4 h and their activity was tested (Figure 7). It has been suggested that when the concentration is less than 400 ug/mL, the cell survival rate is higher than 89%. Especially at a concentration of 200 ug/mL, the cell survival rate was more than 99%. Combined with Figure 8, the luminescence intensity of rare earth, 200 μg/mL concentration of rare-earth nanomaterials not only have sufficient safety but also have a high luminous intensity. When the concentration of rare-earth ions is as high as 500 μg/mL or even 600 μg/mL, the cell survival rate is still higher than 80%. However, no matter how low the concentration of this material is without being modified by DSPE-PEG_2K_, there is always high cytotoxicity. If such material is used for animal experiments, it will cause serious damage to other animal tissues, so the surface must be modified. Furthermore, if Ag-NaYF_4_:Yb^3+^/Er^3+^ is directly modified with DSPE-PEG_2K_, it still does not play a very good role, indicating that DSPE-PEG_2K_ cannot well modify the surface of Ag-NaYF_4_:Yb^3+^/Er^3+^, which also indicates the need for preparing core-shell structure Ag-NaYF_4_:Yb^3+^/Er^3+^@ NaYF_4_:Nd^3+^@NaGdF_4_.

**Figure 9:**
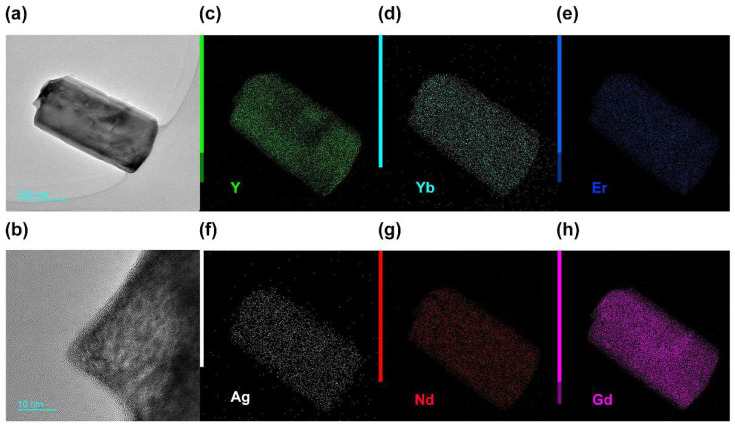
(**a**) TEM of Ag-NaYF_4_:Yb^3+^/Er^3+^@ NaYF_4_:Nd^3+^@NaGdF_4_, (**b**) the high-resolution TEM (HRTEM) image of the surface of Ag-NaYF_4_:Yb^3+^/Er^3+^@ NaYF_4_:Nd^3+^@NaGdF_4_, (**c**–**h**) EDS for (**a**).

To complete this study, TEM and EDS were used to prove the structure (Figure 9). The surface of Ag-NaYF_4_:Yb^3+^/Er^3+^ is covered with a thin layer of NaYF_4_:Nd^3+^, and the surface of NaYF_4_: Nd^3+^ is covered by a thin layer of NaGdF_4_. DSPE-PEG_2K_ can easily modify its appearance.

**Figure 10:**
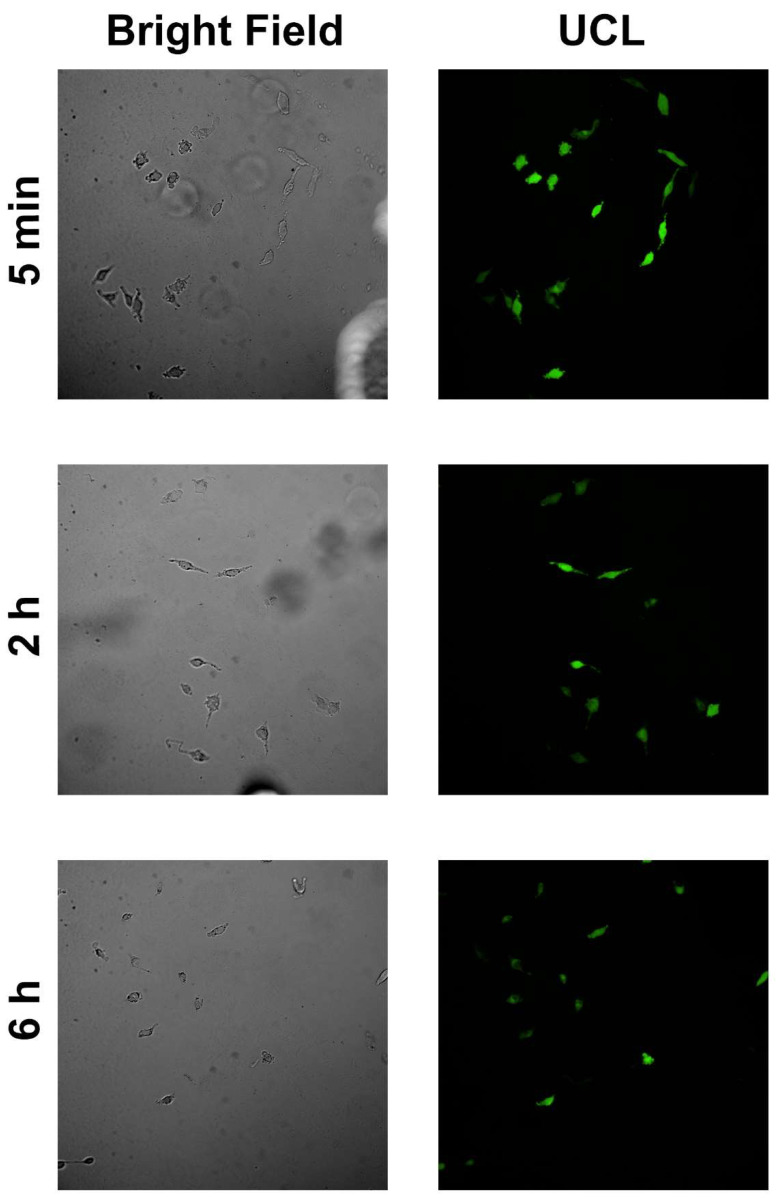
UCL images of HeLa cells incubated with Ag-NaYF_4_:Yb^3+^/Er^3+^@ NaYF_4_:Nd^3+^@NaGdF_4_-DSPE-PEG_2K_ for 5 min, 2 h and 6 h.

**Figure 11:**
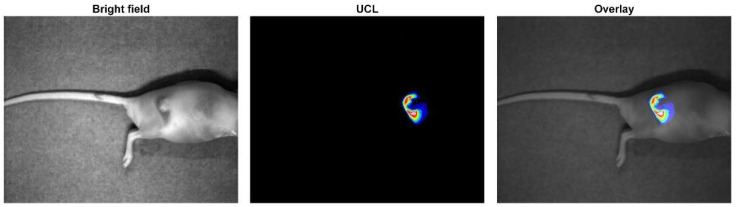
In vivo imaging of a tumor-bearing Balb/c mouse after injection of Ag-NaYF_4_:Yb^3+^/Er^3+^@ NaYF_4_:Nd^3+^@NaGdF_4_-DSPE-PEG_2K_ at the tumor site.

All the characterization of Ag-NaYF_4_:Yb^3+^/Er^3+^@ NaYF_4_:Nd^3+^@NaGdF_4_-DSPE-PEG_2K_ is over, the following is the effect of applying the material to UCL imaging. UCL imaging (Figure 10) yields clear cells makers at an excitation wavelength of 980 nm. The figure shows that after 5 min of incubation, there is light source signal on the cells, and after 2 h even 6 h, the light source signal still exists, indicating that the sample that was easily absorbed by the HeLa cells and can last for a long time. A 200 μg/mL concentration of Ag-NaYF_4_:Yb^3+^/Er^3+^@ NaYF_4_:Nd^3+^@NaGdF_4_-DSPE-PEG_2K_ was injected intratumorally into mice. In vivo imaging (Figure 11) yields clear tumor makers at an excitation wavelength of 980 nm. The figure shows that after 180 min of incubation, there is no other light source signal besides the tumor, indicating that the sample that was injected into the body has no outflow. It also shows that the sample prepared by the CHSS method can be used for tumor imaging.

**Figure 12:**
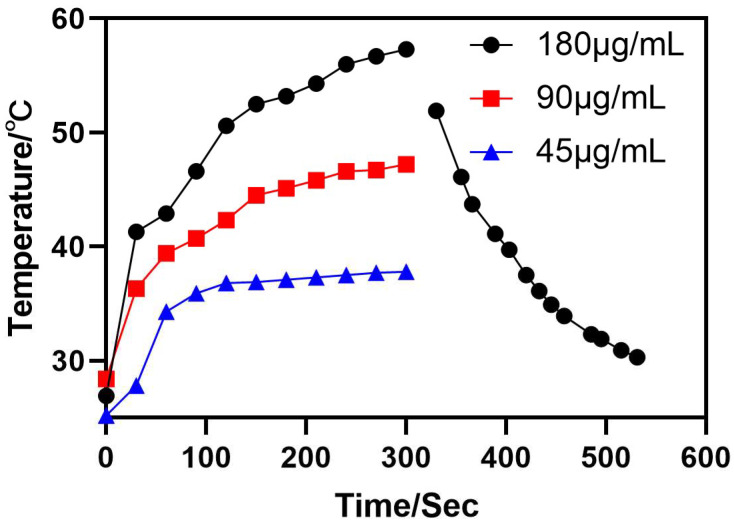
Temperature rise-drop curve of Ag-NaYF_4_:Yb^3+^/Er^3+^@ NaYF_4_:Nd^3+^@NaGdF_4_-DSPE-PEG_2K_ with different concentrations irradiated by 980 nm near-infrared light.

In addition to the imaging function, Ag-NaYF_4_:Yb^3+^/Er^3+^ has a certain photothermal conversion efficiency, and its temperature rise curve (Figure 12) meets the requirements of photothermal treatment. Ag-NaYF_4_:Yb^3+^/Er^3+^ with 180 µg/mL are heated to 57 °C and immediately cooled once the irradiation is stopped. It is commonly known that near-infrared light irradiation at 980 nm wavelength, NaYF_4_:Yb^3+^/Er^3+^ can only generate green visible light without producing heat. However, after doping with silver nanoparticles, it can not only emit brighter visible light as emitting before doping the silver nanoparticles, but also emit heat. The reasons for this phenomenon are as follows: Simultaneously, silver nanoparticles enhance the luminescence intensity of NaYF_4_:Yb^3+^/Er^3+^ under the near-infrared light with the wavelength of 980 nm, so that NaYF_4_:Yb^3+^/Er^3+^ emit stronger light energy with the wavelength of 540 nm. This light energy further excites the silver nanoparticles, resulting in the heat emission of NaYF_4_:Yb^3+^/Er^3+^ doped with silver nanoparticles [23]. In the future, Ag-NaYF_4_:Yb^3+^/Er^3+^@ NaYF_4_:Nd^3+^@NaGdF_4_-DSPE-PEG_2K_ may be used as a reagent for photothermal treatment of cancer.

## 4. Conclusions

Ag-NaYF_4_:Yb^3+^/Er^3+^@ NaYF_4_:Nd^3+^@NaGdF_4_-DSPE-PEG_2K_ was successfully synthesized by the CHSS method. Doping with nanosilver improves the luminous intensity of NaYF_4_:Yb^3+^/Er^3+^, which shows increased luminous intensity by nearly 6.5 times. NaYF_4_:Nd^3+^@NaGdF_4_, which has an autofluorescence effect and is easy to modify, is used as the shell material. When the material concentration is below 600 µg/mL, the cell viability is greater than 90%, and when the concentration is 200 µg/mL for in vivo imaging, the cell viability is greater than 98%, which greatly exceeds the national standard requirement of higher than 87% cell viability, so the overall luminous intensity of the core-shell structure of rare-earth nanomaterials is perfect, and it has high biocompatibility and can be used as an excellent biomedical material in organisms. Second, because of its X-ray attenuation properties and magnetic properties, it can be used in CT and MRI scanning, which combined with fluorescence imaging, will be able to resolve in future multi-mode imaging systems, providing complete and clear images for the diagnosis and treatment of complex cancers such as blood metastasis. Finally, the extremely high luminous intensity of this material is mainly due to the presence of silver nanoparticles, which have good photothermal conversion efficiency which could be used in a photothermal therapy (PTT) combination. In future research, once the targeting problem is resolved, this material could be used to peform the simultaneous diagnosis and treatment of cancer.

## Data Availability

No new data were created or analyzed in this study. Data sharing is not applicable to this article.
